# Loot

**Published:** 1869-05-15

**Authors:** 


### LOOT.

### *Chestnut Leaves in Hooping Cough.*

Dr. J. Ludlow communicates to the Cinn. *Lancet and Observer* the successful results of his treatment of hooping-cough with infusion of chestnut leaves—half-ounce to the pint of boiling water, subsequently diluted with a pint of cold water, sweetened and administered *ad libitum.*

---

### *Obscure Cases of Sudden Death.*

Wooster Beach, Jr., M.D., writes to the *Medical Record:*

Many physicians, and particularly those who make post-mortem examinations for coroners, will frequently meet with cases of sudden death, I might say *instantaneous* death, for which they can discover no satisfactory cause, neither in the symptoms immediately preceding it, nor in the appearance of the body on autopsy. An individual, man, woman, or child, without a premonition of any kind, suddenly utters an exclamation of surprise, perhaps of pain, or it may be, entirely without warning, sinks to the earth. Those near him hasten to his assistance, and find him either entirely dead, or drawing his last breath. In some instances the premonitory symptoms are slightly more marked, or of greater duration, but are not such as to point to the solution of the manner of death. A post mortem examination is made. The body has the external appearance of robust health. Every organ is carefully examined, and found to be in a perfect normal state, and the indications of health are as well marked in the interior as was the case in the exterior of the body. What is the cause of death?

The solution of this question is required, I estimate, in full fifteen per cent. of sudden, or, more properly, instantaneous deaths, and probably in a larger per centage. Without doubt, a great proportion of sudden deaths ascribed to valvular disease of the heart—to uræmia—to serous apoplexy, and to other causes, only occasionally proving suddenly fatal, really belong to this class of obscure causes under consideration. Physicians making examinations, unaware of the frequency of the existence of these causes, naturally ascribe death to a diseased condition, be it ever so slight, of almost any organ.

There are many cases of sudden death, the causes of which become easily apparent on autopsy, as many diseases of the heart, rupture of aneurisms, cerebral apoplexy, etc. I confine myself to those only in which nothing is found to explain the fatal termination; and it is these I designate "obscure cases of sudden death." Some few authors have given attention to this subject; yet it appears to me, without throwing much light upon it, or towards clearing up the mystery that surrounds it. The explanation that has been given in many of these cases, is certainly very unsatisfactory. Some of them I propose briefly to notice.

*Fatty degeneration of the heart.* Too many pathologists of note ascribe death to this disease to make it doubtful that it is a cause. This degeneration of the organ very frequently exists, yet death is seldom traced to it. Sudden deaths, at any rate, not occurring as a rule, in subjects with this affection, should make us exceedingly careful in ascribing such deaths to it even when we find its existence well marked.

*Other affections of the heart* are, of course, very numerous, and I only allude to them to remark that death is undoubtedly referred to them when they are insufficient for its cause. Disease of the heart, according to vulgar and even learned opinion, has to answer for many deaths of which it is entirely innocent. Rheumatism of the heart has been mentioned as terminating suddenly in death, and leaving no traces to show its cause; but we know that rheumatism of the organ pursues a course which gives plain indications of its existence in life, and produces pathological changes easily recognized after death. Paralysis of the heart is said to be accountable for sudden death—but this is a mere hypothesis.

*Embolism* might explain deaths in some instances where the clot is so large as to considerably obstruct the heart's action—but obstruction to a single large vessel of the brain should not be sufficient explanation for death. No more serious results should follow a plug in such vessel, than stopping the flow of blood through it by a ligature.

*Accumulation of flatus in the stomach—fatty degeneration of the diaphragm—engorgement of the lungs and congestion of the brain*, have been given among other causes, but they fail to offer a satisfactory solution of this mode of death. As regards these two latter causes, I may state that it is not unusual to find hypothetic congestion of the lungs, strongly marked, and fullness of the cerebral vessels, but these I consider due to the fluidity of the blood—the blood being almost unexceptionably fluid in these as well as other cases of sudden death.

I have been actuated in bringing the attention of the profession to this subject, by the hope that some suggestion may be elicited as to what manner of inquiry should be adopted in studying this strange phenomenon. Although I have seen a large number of cases of this class, and have had the assistance of able pathologists in their investigation, I am entirely unable to say more in regard to the appearance found, than stated above: that the entire system has presented no deviation from sound health.

Yet some morbid change has taken place as completely and suddenly fatal as an extensive rupture of the heart, or a hemorrhagic effusion around the medulla oblongata.

